# Improved Quadtree-Based Selection of Single Images for 3D Generation

**DOI:** 10.3390/s25216559

**Published:** 2025-10-24

**Authors:** Wanyun Li, Yue Liu, Yuqiang Fang, Yasheng Zhang, Yao Lu, Gege Sun

**Affiliations:** Graduate School, Space Engineering University, Beijing 101416, China; liwanyun@hgd.edu.cn (W.L.); liuyue@hgd.edu.cn (Y.L.); zhangyspublic@163.com (Y.Z.); lybenben@hgd.edu.cn (Y.L.); mxg_0613@outlook.com (G.S.)

**Keywords:** image selection, quadtree structure, 3D generation model, single-view reconstruction

## Abstract

With the rapid development of large generative models for 3D content, image-to-3D and text-to-3D generation has become a major focus in computer vision and graphics. Single-view 3D reconstruction, in particular, offers a convenient and practical solution. However, the way to automatically choose the best image from a large collection to optimize reconstruction quality and efficiency is very important. This paper proposes a novel image selection framework based on multi-feature fusion quadtree structure. Here, we introduce a new image selection method based on a multi-feature quadtree structure. Our approach integrates various visual and semantic features and uses a hierarchical quadtree to efficiently evaluate image content. This allows us to identify the most informative and reconstruction-friendly image from large datasets. We then use Tencent’s Hunyuan 3D model to verify that the selected image improves reconstruction performance. Experimental results show that our method outperforms existing approaches across key metrics. Baseline methods achieved average scores of 6.357 in Accuracy, 6.967 in Completeness, and 6.662 Overall. Our method reduced these to 4.238, 5.166, and 4.702, corresponding to an average error reduction of 29.5%. These results confirm that our approach reduces reconstruction errors, improves geometric consistency, and yields more visually plausible 3D models.

## 1. Introduction

The rapid advancement of generative large models for 3D content creation, particularly in Image-to-3D and Text-to-3D synthesis, has established the automatic generation of 3D models from single-modal inputs (e.g., images or text) as a transformative trend in computer vision and graphics [1,2]. This progress has considerably lowered the barriers to 3D content creation, revealing broad application potential in augmented reality (AR), virtual reality (VR), and digital twins.

Among these approaches, single-view 3D reconstruction has attracted considerable research interest due to its convenient input requirements and practicality, emerging as a key focus in both academia and industry. Recent deep learning-based advances [3,4,5], including DP-AMF [6], Gamba [7], and M3D [8], have made significant strides by developing more powerful neural architectures to infer complex 3D geometry and textures from a single 2D image.

Most existing studies assume that the input image is of high quality. These methods focus on how to reconstruct 3D models, but often ignore a more basic question: “which image should we use?” In practice, users often have many candidate images. These may vary greatly in lighting, angle, occlusion, and clarity—all of which affect reconstruction. Choosing an image arbitrarily can lead to failures, artifacts, or lost details, wasting computational resources.

Therefore, automatically and efficiently selecting the best image from a large set has become a critical but understudied problem. Common methods, such as those based only on image variance, fail to fully capture texture richness, geometric structure, or detail integrity. Traditional quadtree methods use pixel variance to measure local complexity, but their dependence on a single feature makes them unreliable in complex scenes.

To overcome these limitations, we propose a multi-feature quadtree method for image selection. Our approach combines several visual features—pixel variance, edge density, and gradient direction consistency. This allows for a more comprehensive evaluation of image content. The goal is to automatically select the best image from a large collection for high-quality 3D reconstruction. We then use Tencent’s Hunyuan 3D 2.1 model to verify that our selected images improve results.

The main contributions of this paper are as follows:

1. We introduce a novel quadtree model that incorporates multiple visual features—including region size, pixel variance, edge density, and gradient direction consistency—into a unified splitting criterion. This multi-feature fusion mechanism overcomes the limitations of conventional single-feature quadtree methods, enabling more robust image content analysis.
2. We develop a comprehensive scoring function that quantitatively assesses images along three critical dimensions: information richness, structural complexity, and edge preservation. This metric provides a holistic evaluation of an image’s suitability for 3D reconstruction, improving selection accuracy while mitigating overfitting.
3. We establish a fully automated image screening pipeline and conduct extensive validation on real-world datasets. Results show that our method consistently selects images that enhance the reconstruction quality of Tencent’s Hunyuan 3D model, outperforming existing screening approaches on key metrics such as Accuracy and Completeness.

## 2. Related Work

### 2.1. Quadtree Structure

The quadtree is a classical hierarchical spatial data structure that finds widespread application in computer graphics [9,10], geographic information systems [11,12] and image processing [13,14]. It recursively subdivides a two-dimensional space into four quadrants (child nodes), with each node representing a distinct rectangular region of an image. This process efficiently constructs a tree structure that supports multi-resolution representation of image content.

Traditional image screening methods based on the quadtree typically involve the following steps: (1) Region Segmentation: Starting with the entire image as the root node, the space is recursively partitioned into four uniform quadrant subregions, each forming a child node. (2) Region Characterization: For the image region corresponding to each node, the variance of its pixel values is computed to quantify local complexity or information content. (3) Split Control: A node is further subdivided if its pixel variance exceeds a predefined threshold and its size remains above a minimum partition threshold—indicating that the region contains sufficient detail to warrant finer analysis. (4) Screening Decision: Finally, the overall complexity of the image is evaluated using statistical metrics derived from the tree, such as its maximum depth or the total number of leaf nodes. Images that yield deeper trees or a greater number of nodes are considered more complex and thus deemed more suitable for subsequent processing tasks (e.g., 3D reconstruction). Partial results of this quadtree-based partitioning process are illustrated in the figure below.

**Figure 1:**
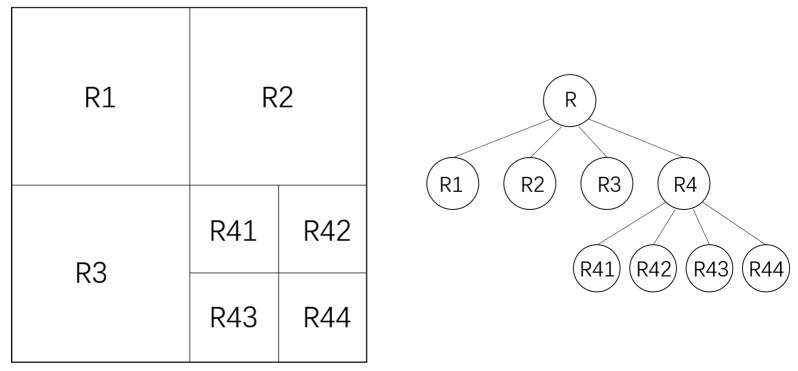
Schematic diagram of a quadtree structure, R means Root Node.

As illustrated in Figure 1, the quadtree algorithm recursively subdivides an image in-to homogeneous regions. However, these methods face a key limitation: they rely only on pixel variance to segment images. This narrow focus fails to capture other important visu-al features, such as texture, shape, and edges. As a result, when images contain fine details, complex patterns, or strong contrasts, such approaches often miss critical information. This leads to unreliable and suboptimal selection, which does not fully reflect the image’s actual complexity or informational value.

### 2.2. Single-View Reconstruction

Single-view 3D reconstruction uses just one image as input, offering a convenient and efficient way to create 3D models. This practicality has made it a popular research topic. However, recovering 3D shape from a single image is fundamentally challenging because the process is ill-posed, key depth and scale cues are missing. Recent advances use deep learning to improve reconstruction. New neural network designs help infer geometry and texture from 2D images.

However, single RGB images lack explicit scale and depth information, often leading to blurred or distorted reconstruction outcomes, particularly in occluded or textureless regions. To address these challenges, Zhang et al. proposed DP-AMF [6], a method that introduces a depth-prior-guided adaptive multi-modal fusion mechanism. This approach integrates global and local feature fusion strategies, and the refined features are subsequently passed to an SDF decoder to generate high-quality 3D meshes.

With the growing demand for automated 3D content generation pipelines, single-view reconstruction still faces substantial memory consumption issues. Shen et al. developed a sequential modeling network named Gamba [7], which leverages contextual reasoning capabilities and linear scalability in token length. This architecture supports large-scale 3D Gaussian splatting, thereby advancing single-view reconstruction based on Gaussian representations.

Another challenge is accurately rebuilding objects in complex scenes. Many current methods fail to balance global structure and local detail. A team from the University of Tsukuba and The Hong Kong Polytechnic University introduced M3D [8], which uses a dual-stream network to merge global and local features. It also incorporates depth estimation, helping blend visual and geometric cues. This approach improves accuracy and preserves fine details, delivering state-of-the-art performance.

Conventional single-view 3D reconstruction methods often rely on complex multi-view geometry principles. Most approaches require large amounts of annotated data and involve sophisticated network architectures. Although some studies have introduced techniques such as depth priors and multi-scale feature fusion to improve reconstruction quality, these methods often increase system complexity and implementation difficulty. Moreover, they still struggle to fully compensate for the inherent lack of geometric and depth information in single-view images. As a result, generative models are now being considered to address such fundamental issues as missing geometry and incomplete shape inference.

### 2.3. AI-Based 3D Model Generation

In recent years, AI-based 3D content generation has started to change how digital assets are made. By automating production, these tools help more people create 3D models with less time and effort. Most modern 3D generative systems include two core parts: (1) a 3D variational autoencoder (3D-VAE) that encodes 3D shapes into a compact latent form and decodes them back; (2) a diffusion model that operates in this latent space, generating new 3D models from text or image inputs.

Building on this framework, a research team at The Hong Kong University of Science and Technology, led by Ping Tan, developed the Dora model [15]. Dora incorporates a salient edge sampling method and a dual cross-attention mechanism. This improves both reconstruction quality and compression performance. During training, the system identifies regions with high geometric detail and processes them with higher priority. This helps preserve fine features that are often lost with standard uniform sampling.

Following the 3D-VAE training paradigm, CraftsMan3D [16] uses a two-stage process inspired by conventional modeling. First, a 3D diffusion model produces initial shapes and textures from multi-view images. Then, a refinement stage uses super-resolution normal maps and differentiable rendering to enhance geometric details.

A team from Tsinghua University introduced Trellis [17]. This method uses a structured latent representation (SLAT) attached to voxels near object surfaces. These features are extracted from rendered views using a visual encoder, capturing both structure and appearance. Different decoders can then be applied to map the SLAT representation into various high-quality 3D formats.

Other notable contributions include Tencent’s Hunyuan 3D [18], a platform that accepts both text and image inputs; VAST AI’s TripoSR [19], which uses a transformer for fast 3D generation; and Yingmu Technology’s Rodin [20], which builds 3D scenes with neural radiance fields [21]. These approaches provide diverse and effective solutions for high-quality 3D content creation. Current 3D generation methods often suffer from high computational costs and limited generalization capabilities. Tencent Hunyuan 3D addresses these issues by supporting multimodal inputs and employing an efficient end-to-end architecture, significantly improving practicality and deployment efficiency while maintaining high output quality.

## 3. Method

### 3.1. Image Screening Framework Based on Multi-Feature Fusion and Quadtree

The proposed method employs a multi-feature split criterion—integrating region size, pixel variance, edge density, and gradient direction consistency to identify images that yield optimal 3D reconstruction performance from large-scale datasets of specific objects. This approach enhances the accuracy and robustness of 3D reconstruction while significantly reducing computational costs.

The process initiates by constructing a quadtree for each image, where the entire image forms the root node. For each node, we compute its variance, edge density, and gradient direction consistency. The latter is quantified by dividing the gradient orientations into a predefined number of bins and calculating the proportion of the most frequent orientation. A node is subdivided only if its size and all three feature values simultaneously exceed their respective thresholds. This splitting process continues recursively until no more nodes satisfy the condition. Subsequently, the method traverses all leaf nodes of the fully constructed quadtree to aggregate their statistical features. A comprehensive quality score is then computed based on these aggregated features. Finally, images are ranked in descending order of their scores, and the top-K images are selected for subsequent 3D reconstruction tasks.

**Figure 2:**
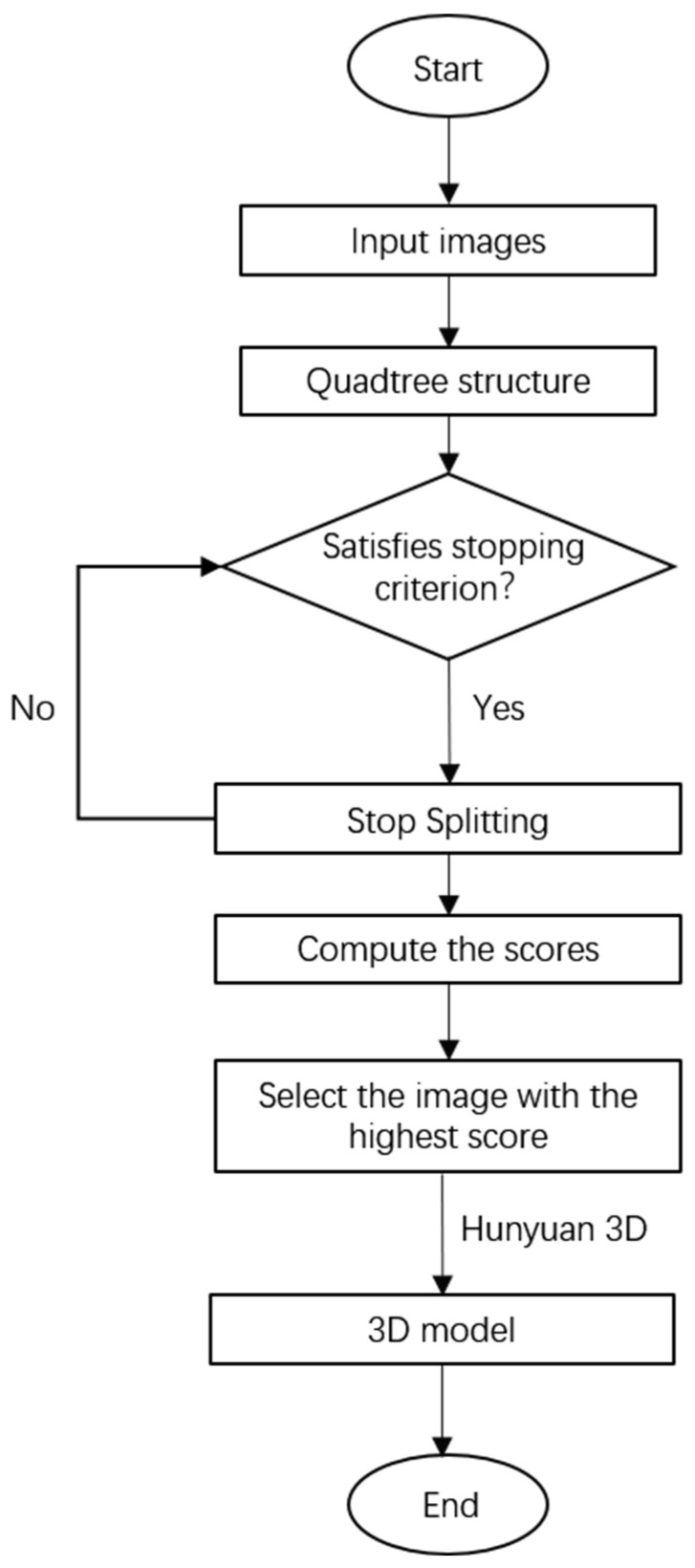
Flowchart of the multi-feature fusion quadtree image screening method.

Figure 2 presents a flowchart of the image screening method based on multi-feature fusion quadtree.

In our method, the variance, edge density, and gradient direction consistency are calculated to quantify local complexity as follows:

(1) $$V\mathrm{ar}(I)=\frac{1}{P}{\sum_{i=1}^{P}\left(x_{i}-\mu \right)}^{2},$$

(2) $$Edge(I)=\frac{1}{P}\sum_{i=1}^{P}\left|\nabla I(x_{i})\right|,$$

(3) $$\theta =\arctan(\frac{G_{y}}{G_{x}}).$$

Among these, Var(I) represents variance, I denotes the current image block; P represents the total number of pixels, i denotes the pixel index, $x_{i}$ denotes the grayscale value of the i th pixel, and μ denotes the mean value of the region’s pixels; Edge(I) represents edge density, $\nabla I(x_{i})$ represents the magnitude of the gradient computed by the Sobel operator, θ represents the gradient direction calculated using the Sobel operator.$G_{x}$ denotes the horizontal gradient component, and $G_{y}$ denotes the vertical gradient component.

After obtaining the $G_{x}$ and $G_{y}$ for one pixel, we use Formula (3) to find its gradient direction. This angle means the image changes the most at that spot. Now we have the gradient directions for many pixels in the image block. The next step is to count how these directions are spread out. We make a histogram by dividing the directions into groups and see which group has the most pixels. This helps us find the main direction and see how consistent the directions are.

The method for dividing the gradient direction into a preset number of intervals, calculating the proportion of the dominant direction, and obtaining the direction consistency value is as follows:

(4) $$\gamma =\frac{\mathrm{Maximum}\;\mathrm{Count}\;\mathrm{Interval}\;\mathrm{in}\;\mathrm{Directional}\;\mathrm{Histogram}}{\mathrm{Total}\;\mathrm{edge}\;\mathrm{pixel}\;\mathrm{value}}.$$

In the formula, γ represents the direction consistency value. The maximum count interval in the direction histogram is determined by dividing the gradient direction range $\theta =0\sim 180^{\circ }$ into 8 direction histogram count intervals, each spaced at 22.5°, and then identifying the interval with the highest count.

The segmentation threshold based on this method is:

(5) $$\left\{\begin{array}{l} \mathrm{Node}\;\mathrm{size}>S_{\min},\\ \mathrm{Variance}\geq \alpha \cdot {\sigma_{0}}^{2},\\ \mathrm{Edge}\;\mathrm{Density}\geq \beta ,\\ \mathrm{Direction}\;\mathrm{Consistency}\;\mathrm{Value}\geq \gamma ’ \end{array}\right..$$

In the formula, node size denotes the dimensions of the current node image block I; $S_{\min}$ represents the minimum segmentation size, α denotes the variance constant, ${\sigma_{0}}^{2}$ represents the initial variance of the whole image, β denotes the edge density threshold, and γ’ denotes the direction consistency threshold.

The root node of the quaternary tree splits into four child nodes, corresponding to the northwest (NW), northeast (NE), southwest (SW), and southeast (SE) quadrants. Each child node continues to divide into four child nodes until terminal nodes matching task requirements are obtained, marked as leaf nodes, at which point splitting ceases. The collected statistical features are: $F=[N,D_{\max},\bar{\rho }]$; where F denotes the statistical feature; N represents the total number of splits in the quaternary tree; $D_{\max}$ is the maximum depth; $\bar{\rho }$ is the average edge density.

The comprehensive score for statistical features is calculated as:

(6) $$score=0.5\times D_{\max}+0.3\times N+0.2\times \bar{\rho }.$$

The maximum depth is calculated as follows: $D_{\max}=\log_{2}(\frac{\min(W,H)}{S_{\min}})$; (W,H) represents node size, W denotes width, and H indicates height. The maximum depth weight reflects information richness—greater depth indicates more complex local information. In order to determine the optimal parameter configuration, we adopted a rigorous experimental strategy. First, we defined a multidimensional parameter space containing all key hyperparameters, such as the node count weight and edge density weight. We set reasonable value ranges for each parameter based on domain knowledge. We then abandoned computationally expensive grid search. Instead, we used a random search-based hyperparameter optimization strategy. This method efficiently samples a large number of parameter combinations at random from the predefined parameter space. Each combination was evaluated individually for its reconstruction performance on a separate validation set. The node count weight of 0.3 prevents overfitting due to excessive nodes. The average edge density weight of 0.2 aids validation by preserving regions with high edge density. Directional consistency is already incorporated into the splitting criteria, ensuring retained nodes inherently possess directional coherence. Re-evaluating disordered nodes would introduce redundant computation. Introducing a fourth scoring dimension could increase overfitting risk. Instead, utilizing maximum depth, node count, and average edge density for final scoring enables global differentiation between detail richness and structural complexity while enhancing computational efficiency. Images with the highest composite scores are employed as input for subsequent 3D reconstruction tasks.

### 3.2. Hunyuan3D 2.1

Here we employ the Tencent Hunyuan 3D 2.1 [18] to perform 3D reconstruction on the selected images. Building upon versions 1.0 [22] and 2.0 [23], Hunyuan 3D 2.1 represents a further optimized and scalable 3D asset creation system. It advances 3D generation technology through two key innovations: a fully open-source framework and physically based rendering texture synthesis. As shown in Figure 2, the model comprises an autoencoder (3D-ShapeVAE) and a flow-based diffusion model (3D-DiT). 3D-ShapeVAE compresses 3D assets represented by polygon meshes into continuous token sequences in latent space. 3D-DiT trains on this latent space to predict token sequences for input images, which are then decoded back to polygon meshes via the VAE decoder.

3D-ShapeVAE utilizes the vector sets introduced by 3Dshape2VecSet [24], then employs a variational autoencoder-decoder (3D-ShapeVAE). For input meshes, the surface point cloud undergoes uniform sampling and importance sampling. The encoding process first applies Farthest Point Sampling (FPS) to and, generating query points. These points are then connected to form the final point cloud $P\in R^{(M+N)\times 3}$ and query set $Q\in R^{(M^{\prime }+N^{\prime })\times 3}$. Both and undergo Fourier position encoding followed by linear projection, yielding encoded features $X_{p}\in R^{(M+N)\times d}$ and $X_{q}\in R^{(M^{\prime }+N^{\prime })\times d}$, where d is the dimension. Within the variational autoencoder framework, the latent shape embedding $Z_{s}\in R^{(M^{\prime }+N^{\prime })\times d_{0}}$ is predicted, where $d_{0}$ represents the latent dimension. The decoder reconstructs a 3D neural field from the latent shape embedding, ultimately converting it into a Signed Distance Function (SDF), which is then transformed into a triangular mesh via Marching Cubes. During network training, reconstruction loss and KL-divergence loss supervise model training. Reconstruction loss calculates the MSE between predicted $SDFD_{s}(\left.x\right|Z_{s})$ and ground truth SDF(x), while ensures compactness and continuity in the latent space. The total loss formula is:

(7) $$L_{r}=E_{x\in R^{3}}[MSE(Ds(\left.x\right|Z_{s}),SDF(x))]+\gamma L_{KL},$$

where γ is a constant.

**Figure 3:**
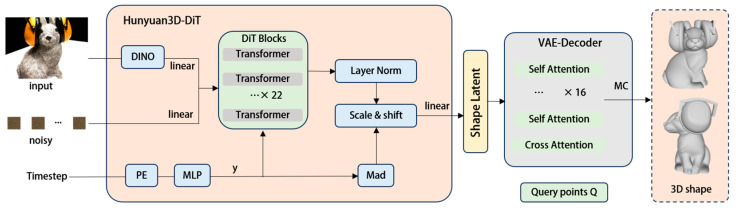
Hunyuan3D pipeline for shape generation. Given a single image input, combining Hunyuan3D-DiTand Hunyuan3D-VAE can generate a high-quality and high-fidelity 3D shape.

Figure 3 illustrates the pipeline of Hunyuan3D for shape generation. As shown, given a single image input, the synergistic integration of Hunyuan3D-DiT and Hunyuan3D-VAE enables the generation of a high-quality and high-fidelity 3D shape. Simultaneously, Hybrid 3D 2.1 introduces a PBR material texture synthesis framework that transcends traditional RGB texture mapping. To accurately describe the surface reflectance characteristics of generated 3D assets and precisely simulate the distribution of geometric micro-surfaces, it also incorporates 3D-Aware RoPE [25] to focus on spatial information. This architecture implements the Disney Principled BRDF model [26] to generate high-quality PBR material maps. It retains ReferenceNet’s reference image feature injection mechanism while connecting the normal map and canonical coordinate map (CCM) from geometric rendering to latent noise.

In summary, Tencent Hunyuan 3D 2.1 presents a unified, open-source framework that integrates high-fidelity geometry generation with physically based rendering (PBR) material synthesis, offering a comprehensive and innovative solution for 3D content creation. The architecture synergistically combines a diffusion transformer (DiT)-based geometry generator and a physics-aware material module, enabling efficient production of 3D assets characterized by both rich geometric detail and high material realism. By open-sourcing the complete pipeline—including data processing procedures, training frameworks, and pre-trained model weights—the system greatly improves accessibility and reproducibility, thereby promoting broader adoption and advancement in the field of 3D generation.

## 4. Experiment and Discussion

### 4.1. Test Design

This experiment was conducted on a Windows 11 operating system using Python 3.11.0 and the PyTorch 2.1.0 framework with CUDA 11.8 support. The hardware configuration consisted of a 12th Gen Intel^®^ Core™ i7-12700H processor (Intel, Santa Clara, CA, USA) and an NVIDIA GeForce RTX 3060 Laptop GPU (NVIDIA, Santa Clara, CA, USA) with 6 GB of VRAM, accompanied by 24 GB of system RAM. The experimental data were obtained from the widely adopted DTU dataset [27], a standard benchmark for 3D reconstruction tasks. This dataset employed high-precision industrial-grade 3D scanners to conduct comprehensive scans of multiple scenes, acquiring a substantial volume of high-resolution point cloud data. This data serves as the ground truth for subsequent evaluation. We choose ten representative scenes, covering a variety of object types including beverage containers, toy models, fruits, and kettles. Each scene contained either 49 or 64 multi-view images, each with a resolution of 1600 × 1200 pixels. Based on numerical analysis of image patches from the DTU dataset and randomized search on the validation set, parameter settings were applied: min_size was set to 16 pixels to terminate recursion at appropriately small regions; var_threshold was set to 100 to prevent excessive splitting in texture-less areas; both edge_threshold and direction_threshold were set to 0.03 to preserve salient edges and enforce directional consistency.

To systematically evaluate the effectiveness of the proposed method in 3D reconstruction tasks, we conducted comparative experiments using the traditional quadtree-based filtering approach as a baseline. The reconstruction quality under different input filtering strategies was assessed using standard quantitative metrics for surface reconstruction, including accuracy, completeness, and overall score, to provide a comprehensive evaluation of the performance.

Accuracy quantifies the deviation of the reconstructed points from the ground truth surface and is defined as follows:

(8) $$accuracy(d)=\left\{p\in P:\min_{g\in G}\left\|p-g\right\|\right\},$$

Among these, P is the set of predicted points sampled from the reconstructed grid, G is the set of ground truth points.

Completeness, as a complementary metric to accuracy, evaluates the extent to which the true surface is captured by the reconstruction. It is defined as the distance from the ground truth surface to the reconstructed point cloud, and is computed as follows:

(9) $$completeness(d)=\left\{g\in G:\min_{p\in P}\left\|g-p\right\|\right\},$$

After calculating the aforementioned metrics, the overall measure of accuracy and completeness can be computed.

(10) $$overall=\frac{acc+comp}{2}.$$

### 4.2. Evaluation and Analysis

In Table 1, we present a comparative evaluation of the 3D reconstruction performance on the DTU dataset using three key metrics: accuracy, completeness, and overall score. The results demonstrate that, compared to the traditional quadtree-based selection method, our approach achieves a superior overall score, reflecting higher reconstruction accuracy and more complete geometric recovery.

The experimental results indicate that when only a single image is available as input, the coverage of scene information and the richness of spatial representation remain limited, leading to inaccuracies in the geometric position and absolute scale estimation of the reconstructed model. From an average performance perspective, the proposed method exhibits a substantial improvement over conventional approaches across all major evaluation metrics. As summarized in Table 1, the proposed method achieves a notable reduction in reconstruction error across all key metrics. Traditional methods yielded average values of 6.357 (Accuracy), 6.967 (Completeness), and 6.662 (Overall), while our approach significantly lowered these to 4.238, 5.166, and 4.702, respectively—representing an average error reduction of 29.5%. More importantly, a fine-grained analysis of individual cases reveals even more pronounced improvements under challenging conditions. For instance, in the Scan114 scene—characterized by complex occlusions and sparse textures—our method reduced Acc from 6.935 to 2.093, Comp from 4.743 to 2.560, and the Overall metric from 4.550 to 2.327, corresponding to an approximately 49% error reduction. This suggests that our image selection strategy is particularly effective in scenarios where conventional methods struggle most, primarily through better preservation of structural edges and more stable region sampling.

While absolute scale accuracy remains challenging due to the inherent spatial ambiguity of monocular inputs, our method consistently produces more geometrically stable and visually plausible reconstructions. This improvement can be attributed to two key factors: (1) the multi-feature quadtree mechanism enables more informed selection of images with richer geometric cues, and (2) the selected images provide stronger structural priors to the downstream Hunyuan 3D model.

**Figure 4:**
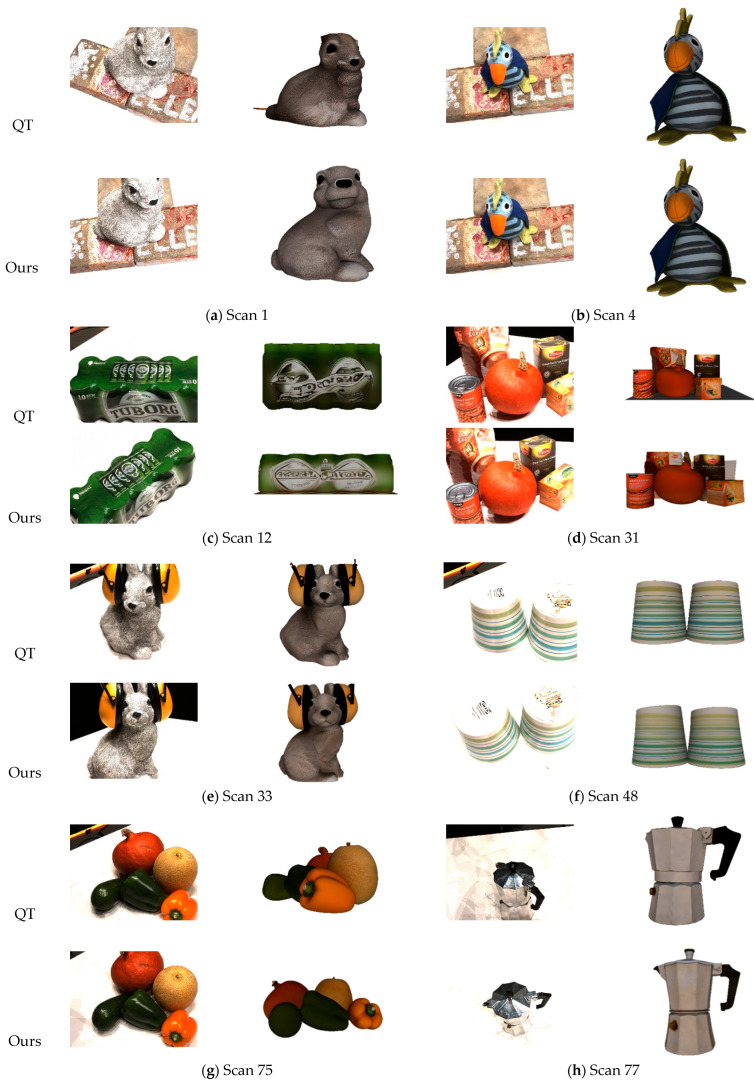

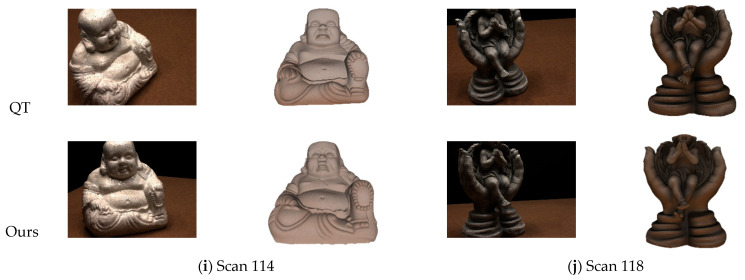
Qualitative comparison of input images and 3D reconstruction results between the traditional quadtree method and the proposed multi-feature quadtree method.

Utilizing Tencent Hunyuan 3D large-scale generative model facilitates not only the efficient synthesis of high-fidelity 3D geometries but also accurate reconstruction of surface textures and material properties. Figure 4 presents a comparative evaluation of reconstruction results across ten diverse scenarios, contrasting the conventional method with the proposed image selection approach. For each scene, the top row displays the input image selected by the baseline method and its corresponding reconstruction, while the bottom row shows the image chosen by our method along with the resulting 3D model. The results clearly demonstrate that the proposed method more effectively identifies images that adhere to a standard front-view perspective. When utilized as input to the Hunyuan model, these images yield reconstructions with superior geometric consistency and visual quality.

As a result, the proposed approach is especially suitable for application contexts that prioritize structural integrity and visual usability over strict metric precision, such as virtual reality, game asset design, conceptual modeling, and rapid prototyping. Future work will focus on integrating multimodal priors and explicit structural constraints to further improve reconstruction accuracy, particularly for scale-critical applications.

In Figure 4, specify in the caption that it compares the traditional quadtree method and the proposed multi-feature quadtree method. Although both methods achieve comparable performance in texture and color reproduction, the proposed approach demonstrates markedly superior geometric accuracy. For instance, in Scans 33 and 114, conventional methods frequently selected images with oblique viewing angles, resulting in inaccurate scale estimation and geometric distortions during reconstruction. In contrast, our method consistently identifies images approximating canonical front-view perspectives, which better satisfy the preferred input conditions for generative 3D models. Consequently, reconstructions derived from our selected inputs exhibit enhanced geometric consistency and structural integrity.

### 4.3. Ablation Experiment

To evaluate the contribution of each component in the comprehensive scoring formula (Equation (6)) to the final selection outcome, we performed a series of ablation experiments. By systematically removing individual parts of the system, we adopted a controlled variable approach to assess the impact of each element on overall performance. We designed four experimental scenarios: (1) Only the node count (Baseline QT), (2) Node count + maximum depth (Ours-D), (3) Node count + edge density (Ours-E), (4) Node count + maximum depth + edge density (Ours-Full). Table 2 presents the ablation results of proposed multi-feature scoring metric. Here, N represents the total number of splits in the quaternary tree; $D_{\max}$ is the maximum depth; $\bar{\rho }$ is the average edge density.

As shown in Table 2, the use of different parameters in the final scoring metric leads to varied performance outcomes. When only the node count is used as the selection criterion (Baseline QT), the reconstruction quality is the poorest. This indicates that a single feature is insufficient for comprehensively evaluating image complexity and information content, resulting in selected images that lack the necessary detail for high-quality 3D reconstruction.

Introducing the maximum depth feature (Ours-D) significantly reduces the reconstruction error. This demonstrates that this dimension effectively captures local structural complexity within image patches, facilitating the selection of regions with richer geometric details.

The addition of the edge density feature (Ours-E) also improves reconstruction, although its performance is slightly lower than that of Ours-D. This suggests that edge information plays an important role in preserving structural integrity in the reconstructed model.

Our full approach (Ours-Full), which integrates all three features, achieves the best reconstruction performance. This result not only confirms that each feature provides unique and indispensable contributions but also highlights the synergistic effect of multi-feature fusion. Together, they enable a more comprehensive and robust evaluation of image content. The ablation study strongly supports the necessity of each component in the proposed scoring metric (Equation (6)).

## 5. Conclusions

The proposed image selection method combines multiple visual features within a quadtree structure to identify informative and structurally representative images—even those with cluttered backgrounds. This approach substantially improves the quality of 3D reconstruction. Experimental results demonstrate the superiority of the proposed method, which significantly outperforms conventional approaches by reducing key metric errors by an average of 29.5%. This confirms its exceptional efficacy in reducing reconstruction errors and improving geometric consistency.

When used with the Hunyuan 3D model, the selected images also improve texture reconstruction, producing surfaces with convincing material details. Although single-view reconstruction still faces challenges—such as recovering exact scale or very fine geometry—the resulting models show strong overall visual quality, coherent occlusion, and high shape fidelity.

This work demonstrates that a multi-feature quadtree selection strategy can significantly enhance the output of generative 3D models. It is especially useful in applications where visual realism and structural coherence matter more than precise metric accuracy—such as in virtual exhibitions, digital content creation, and rapid prototyping. Future work will focus on adapting and optimizing the proposed method for multi-view input sequences. This adaptation aims to address challenging real-world problems, such as complex occlusions and varying illumination. The ultimate goal is to enhance the robustness and generalizability of the reconstruction system.

## Figures and Tables

**Table 1:** Performance comparison of different image screening strategies on the DTU dataset. (QT means Quadtree. Lower acc ↓, comp ↓, and overall ↓ indicate better performance. Units: mm).

	Scan2			Scan4			Scan12			Scan31			Scan33		
	Acc	Comp	Overall	Acc	Comp	Overall	Acc	Comp	Overall	Acc	Comp	Overall	Acc	Comp	Overall
QT	7.657	7.862	7.759	3.619	**3.716**	**3.667**	8.716	8.970	8.843	8.235	10.459	9.347	6.370	**5.711**	6.040
Ours	**3.369**	**6.480**	**4.924**	**2.859**	6.517	4.688	**7.172**	**6.358**	**6.765**	**4.814**	**7.713**	**6.263**	**4.830**	6.277	**5.554**
	Scan48			Scan75			Scan77			Scan114			Scan118		
	Acc	Comp	Overall	Acc	Comp	Overall	Acc	Comp	Overall	Acc	Comp	Overall	Acc	Comp	Overall
QT	6.356	8.358	7.357	8.303	8.942	8.622	3.862	2.781	3.322	6.935	8.593	7.764	**3.513**	**4.275**	**3.894**
Ours	**4.799**	**3.611**	**4.205**	**5.705**	**4.208**	**4.956**	**3.011**	**2.561**	**2.786**	**2.093**	**2.560**	**2.327**	3.731	5.370	4.551

**Table 2:** Ablation study demonstrating the contribution of each feature component to the overall performance of the proposed scoring metric, bold means better performance.

Method	Acc	Comp	Overall
Baline QT (N)	6.935	8.593	7.764
Ours-D (N + $D_{\max}$)	3.475	4.066	3.770
Ours-E (N + $\bar{\rho }$)	5.623	6.772	6.198
Ours-Full (N + $D_{\max}+\bar{\rho }$$\;+\;\bar{\rho }$)	**2.093**	**2.560**	**2.327**

## Data Availability

Data are contained within the article.
